# A Model for Transition of 5′-Nuclease Domain of DNA Polymerase I from Inert to Active Modes

**DOI:** 10.1371/journal.pone.0016213

**Published:** 2011-01-14

**Authors:** Ping Xie, Jon R. Sayers

**Affiliations:** 1 Key Laboratory of Soft Matter Physics and Beijing National Laboratory for Condensed Matter Physics, Institute of Physics, Chinese Academy of Sciences, Beijing, China; 2 Department of Infection and Immunity, Krebs Institute, University of Sheffield Medical School, Sheffield, United Kingdom; University of Medicine and Dentistry of New Jersey, United States of America

## Abstract

Bacteria contain DNA polymerase I (PolI), a single polypeptide chain consisting of ∼930 residues, possessing DNA-dependent DNA polymerase, 3′-5′ proofreading and 5′-3′ exonuclease (also known as flap endonuclease) activities. PolI is particularly important in the processing of Okazaki fragments generated during lagging strand replication and must ultimately produce a double-stranded substrate with a nick suitable for DNA ligase to seal. PolI's activities must be highly coordinated both temporally and spatially otherwise uncontrolled 5′-nuclease activity could attack a nick and produce extended gaps leading to potentially lethal double-strand breaks. To investigate the mechanism of how PolI efficiently produces these nicks, we present theoretical studies on the dynamics of two possible scenarios or models. In one the flap DNA substrate can transit from the polymerase active site to the 5′-nuclease active site, with the relative position of the two active sites being kept fixed; while the other is that the 5′-nuclease domain can transit from the inactive mode, with the 5′-nuclease active site distant from the cleavage site on the DNA substrate, to the active mode, where the active site and substrate cleavage site are juxtaposed. The theoretical results based on the former scenario are inconsistent with the available experimental data that indicated that the majority of 5′-nucleolytic processing events are carried out by the same PolI molecule that has just extended the upstream primer terminus. By contrast, the theoretical results on the latter model, which is constructed based on available structural studies, are consistent with the experimental data. We thus conclude that the latter model rather than the former one is reasonable to describe the cooperation of the PolI's polymerase and 5′-3′ exonuclease activities. Moreover, predicted results for the latter model are presented.

## Introduction

**Figure 1:**
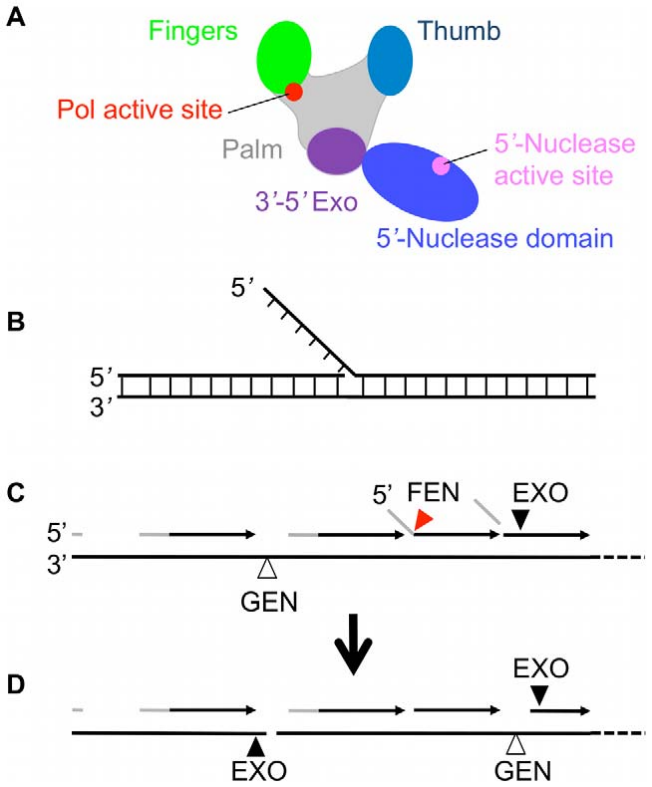
Schematic diagrams of polymerase I and flap DNA. (A) Polymerase I is composed of polymerase domain, which consists of finger, palm, thumb and 3′-5′ exonuclease subdomains, and 5′-nuclease domain. (B) Flap DNA with no single-stranded gap. (C) From left to right. Okazaki fragments consist of a few nucleotides of RNA primer (grey lines), which are then extended by DNA polymerases using deoxynucleside triphosphates to make the daughter DNA strand (dark arrows). Okazaki fragment synthesis gives rise to flap structures as follows when the 3′ end of a newly synthesized strand encounters the 5′ end of the RNA primer of the downstream Okazaki fragment. Strand displacement synthesis occurs (centre right) and the flap is then cleaved by FEN activity (red triangle) of Pol1 5′-nuclease domain. Normally a nick results which is sealed by DNA ligase. (D) Excessive or unregulated FEN EXO or GEN activity (shown by black and open triangles respectively) could give rise to extended single-stranded regions or even double strand breaks as shown.

DNA polymerase I (PolI) is a well-characterized enzyme involved in DNA replication and repair [1]–[4]. The archetypal enzyme (Figure 1A) possesses three distinct biochemical activities, namely a DNA-dependent 5′-3′ DNA polymerase, a 3′-5′ proofreading exonuclease and a 5′-3′ exonuclease function [5], [6]. While polymerase and proofreading functions have been investigated extensively, the 5′-3′ exonuclease activity has been subject to less scrutiny. This activity has been variously described as a 5′-3′ exonuclease, a 5′ nuclease [7] and most commonly as a flap endonuclease or FEN activity [8] due to the biochemical and sequence homologies with their eukaryotic counterparts.

These enzymes have the ability to degrade flap structures, single-stranded and double-stranded DNA from free 5′ ends (Figure 1B, 1C) [9]. The PolI FEN domain processes branched DNA structures such as 5′-flap strands generated when PolI carries out *in vitro* DNA synthesis on circular single-stranded DNA or at a nick within a duplex DNA substrate [7], [10], [11] (Figure 1). These nicks consist of the 3′-hydroxyl group of the last nucleotide added to the chain and the 5′-phosphate group of the next nucleotide on the adjacent chain. Such nicks are sealed efficiently in the presence of DNA ligase and ATP [12].

PolI carries out similar processing of Okazaki fragments during *in vivo* replication of the lagging strand DNA [13],[14]. This process is common to all organisms because the lagging strand is synthesized as a discontinuous series of newly polymerized nucleotides initiated from a short primase-derived RNA primer. The short RNA primer is removed largely by the 5′-3′ exonuclease of PolI in the case of *E. coli*. Although the 5′-3′ exonuclease activity of PolI has long been known to be essential for viability in *Streptococcus pneumoniae* [15], the wider importance of this activity was unclear until 2007 when Fukushima and co-workers presented evidence that either the DNA PolI 5′-nuclease domain or a second paralogous 5′-3′ exonuclease-like protein [16], [17] was essential for cell viability in *E. coli*, *Bacillus subtilis* and *Synechococcus elongates* [18].

Eukaryotes possess multiple homologues of the PolI 5′-3′ exonuclease domain [19], [20] and some of these have been shown to possess very similar biochemical properties to their bacterial counterparts [8], [21], [22]. They too are essential for cell viability as well as playing important roles in replication and repair [23]. Discrete 5′-3′ exonucleases, devoid of polymerase domains are also present in bacteriophages [24] and in some primitive bacteria, e.g. *Mycoplasma* [25]. In all of these cases, the organism's DNA polymerase functions are encoded by completely separate genes. This differs from the majority of cases in eubacterial cells, in which the 5′-3′ exonuclease activity is part of the PolI polypeptide [3].

The consequences of inappropriate Okazaki fragment processing could be catastrophic to the cell (Figure 1C). Should excessive 5′-nuclease activity be displayed then free 5′ ends of Okazaki fragments would suffer extensive degradation leading to larger and more persistent single-stranded gaps being generated in the DNA. These gaps would then need to be filled-in a second time by PolI's polymerase function, using more nucleotide triphosphates than would normally be the case. This would place an excessive and unnecessary energy burden on the cell. However, this is not the worst that could happen. Single-stranded DNA regions are intrinsically more prone to breakage and potentially mutagenic modification than when in the double-stranded form. Furthermore, 5′-nucleases can cleave across the single-stranded region of a gapped DNA structure leading directly to potentially lethal double-strand breaks. The latter activity was named gap endonuclease (GEN) activity by Zheng *et al*. [26] but the reaction had been described earlier in a prokaryotic 5′ nuclease [27]. Clearly, the polymerase and 5′-nuclease catalytic activities of DNA PolI must be coordinated during lagging strand replication for optimum efficiency, fidelity and energy utilization.

### Rigid PolI model: transition of flap DNA from polymerase domain to 5′-nuclease domain

To perform its biological function, the physically linked polymerase and 5′-nuclease domains of PolI (see Figure 1A for schematic diagram of PolI and the corresponding crystal structure shown in Figure S1) must collaborate so as to leave a nick in the flap DNA substrate (see Figure 1B) that can be sealed by DNA ligase. To realize this collaboration two scenarios can be envisaged. The first one is that the relative position of the two domains of PolI is assumed to be fixed (rigid PolI model) while the DNA substrate can transit from its binding position located in the polymerase domain to that located in the 5′-nuclease domain. In the second scenario the DNA substrate keeps its binding position fixed relative to the PolI while the 5′-nuclease domain alters its position relative to the polymerase domain to reposition itself on the scissile site, i.e., the 5′-nuclease active site can transit from a position distant from the cleavage site on the DNA substrate to the site of the scissile phosphate diester bond (flexible PolI model). To determine which one is reasonable, we set out to study the simulated dynamics of the two scenarios. In this section, we consider the first scenario (the rigid PolI model) and study the transition dynamics of the flap DNA substrate from the polymerase domain to 5′-nuclease domain.

**Figure 2:**
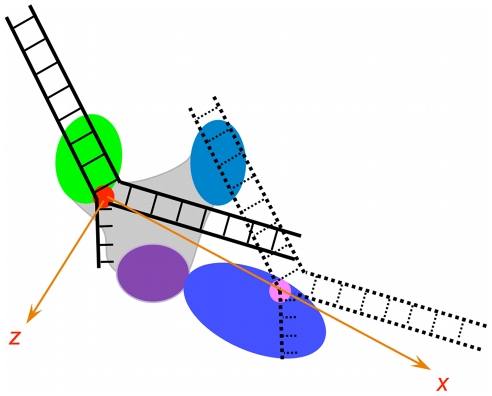
Schematic illustrations of flap DNA transition from PolI's polymerase active site to 5′-nuclease active site.

It is hypothesized that the 5′-nuclease domain has a high affinity for the flap DNA substrate having no single-stranded gap (Figure 1B), which is consistent with experimental data [7], [28]–[30]. Based on this hypothesis, the rigid PolI model is schematically shown in Figure 2. Consider that the polymerase domain has just synthesized a transient flap, with the DNA substrate binding to the polymerase domain, where the polymerase active site is positioned at the fork of the flap (see DNA substrate with solid lines in Figure 2). Due to thermal noise, the flap DNA substrate detaches from the binding site in the polymerase domain. Then, the flap DNA substrate either diffuses freely into solution or diffuses towards the binding site in the 5′-nuclease domain to which it then binds (see DNA substrate with broken lines in Figure 2).

Now, based on the model we carried out a quantitative study of the dynamics of DNA transition. Take the coordinate O*xyz* as shown in Figure 2, where the origin O is taken at the position of the polymerase active site and the *y* axis is perpendicular to the paper surface. The interaction potential of the DNA substrate with the polymerase domain and the 5′-nuclease domain can be written as
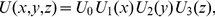
(1)where *U*_0_ is the interaction strength of the DNA substrate with the polymerase domain or the 5′-nuclease domain. *U*_1_(*x*), *U*_2_(*y*) and *U*_3_(*z*) can be written in the following forms
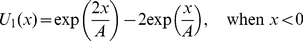
(2)

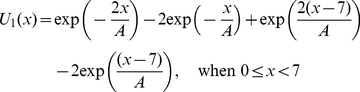
(3)

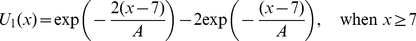
(4)

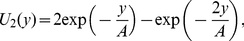
(5)

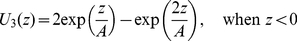
(6)

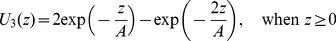
(7)where the polymerase active site is positioned at (*x*, *y*, *z*)  =  (0, 0, 0) while the 5′-nuclease active site is positioned at (*x*, *y*, *z*)  =  (7 nm, 0, 0). The parameter *A* characterizes the interaction distance of the potential. For clarity, the forms of *U* (*x*, 0, 0), *U* (0, *y*, 0) and *U* (0, 0, *z*) are shown in Figure S2. The forms of the potential given in Eqs. (2)–(7) are similar to the Morse potential that characterizes van der Waals interactions. Here, we examine the potentials having the forms given in Eqs. (2)–(7) in our calculations. It is known that the dynamics of a Brownian particle escaping from a potential well depend mainly on the well depth of the potential but is insensitive to the form of that potential (see. e.g., Ref. [31]). Thus, the calculated results presented in this work depend mainly on values of the well depth of the potential while forms of the potential are not important. For example, another form of the potential *U*(*x*, *y*, *z*) gave only slightly different statistical results but has no effect on our conclusion (see below).

The movement of the DNA substrate relative to the PolI in viscous solution can be described by Langevin equations:
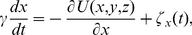
(8)

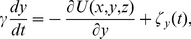
(9)

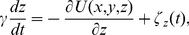
(10)where *γ* is the frictional drag coefficient on the DNA and 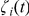 (*i* =  *x*,*y*,*z*) is the fluctuating Langevin force with 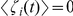 and 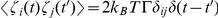. Since calculation of the drag coefficient on an irregularly shaped object such as a flap DNA is complex, for simplicity, we take the drag coefficient of the flap DNA to be 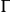 = 5.65
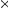
10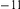 kg

s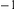, which is equivalent to the drag coefficient 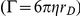 on a sphere with radius of *r_D_* = 3 nm in a solution with viscosity of *η* = 0.01 g

cm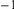 
s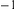 at T = 298 K.

**Figure 3:**
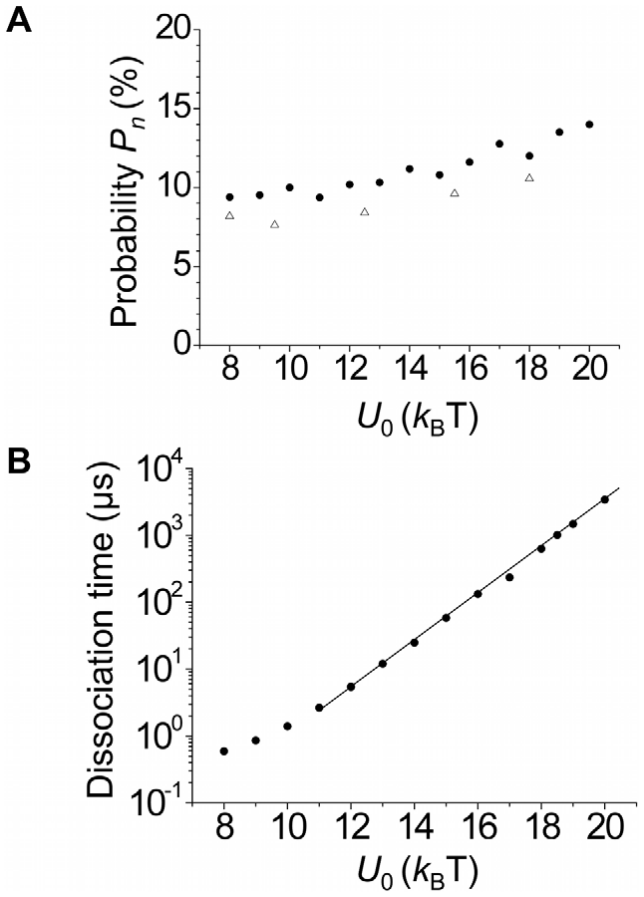
Calculated results on dynamics of flap DNA transition from PolI's polymerase active site to 5′-nuclease active site or dissociation into solution. (A) Probability, *P_n_*, for the DNA to transit to the 5′-nuclease active site versus the interaction strength *U*_0_. (B) Mean time, *T_d_*, for the DNA to detach from the polymerase or to transfer to the 5′-nuclease active site versus the interaction strength *U*_0_. Filled dots represent results with potentials given by Eqs. (2)–(7), with forms of *U* (*x*, 0, 0), *U* (0, *y*, 0) and *U* (0, 0, *z*) shown in Figure S2; while unfilled triangles represent results with forms of potentials *U* (*x*, 0, 0), *U* (0, *y*, 0) and *U* (0, 0, *z*) shown in Figure S7.

To study the transition dynamics of the flap DNA substrate, we solved Eqs. (8)–(10) numerically by using the stochastic Runge-Kutta method [32], [33]. The method has been proved suitable for simulation of stochastic dynamics in physical, chemical and biological systems [32]–[35]. In our simulation, we take *A* = 0.5 nm as is consistent with the Debye length in the order of 1 nm in solution (see Figure S2). In Figures S3, S4, S5, S6 we show some typical results for the trace of the flap DNA substrate relative to the polymerase, where Figure S3 corresponds to the situation where the DNA is transferred from the polymerase active site at (*x*, *y*, *z*)  =  (0, 0, 0) to the 5′-nuclease active site at (*x*, *y*, *z*)  =  (7 nm, 0, 0), while Figsure S4–S6 correspond to the case where the DNA detaches from the polymerase or dissociates into solution. Note that the different traces shown in Figures S3, S4, S5, S6 correspond to different thermal noise realizations. The statistical results of the probability, *P_n_*, for the DNA to transfer to the 5′-nuclease active site versus the interaction strength *U*_0_ are shown in Figure 3A (denoted by dots). The corresponding probability for the DNA to dissociate into solution is thus given by 1 – *P_n_*. The statistical results of the mean time, *T_d_*, for the DNA to detach from the polymerase (i.e., to move to position satisfying 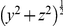 ≥10 nm) or to move to the 5′-nuclease active site at (*x*, *y*, *z*)  =  (7 nm, 0, 0) versus *U*_0_ are shown in Figure 3B. To see the effect of the potential forms, in Figure 3A. We also show some results (denoted by triangles) resulting from use of another form of the potential *U*(*x*, *y*, *z*) such as that plotted in Figure S7. It is seen that different potential forms only give slightly different statistical results.

From Figure 3, it is seen that *P_n_* increases only slowly with the increase of *U*_0_ while *T_d_* increases significantly with the increase of *U*_0_. When *U*_0_>11 *k*_B_T, *T_d_* increases exponentially with *U*_0_. From Figure 3A it is interesting to note that, even for a large value of *U*_0_ = 20 *k*_B_T, the probability *P_n_* that the DNA substrate transfers from the polymerase active site to the 5′-nuclease active site of the same PolI is only slightly larger than 0.1, whereas the probability for the DNA to dissociate into solution is nearly 0.9. This implies that only a minority of 5′-nucleolytic processing events are carried out by the same PolI molecule that has just extended the upstream primer terminus. In other words, in order to cleave the flap, the DNA substrate has to first dissociate from the polymerase domain of the PolI molecule that has just synthesized the flap and then bind to the 5′-nuclease domain of another PolI molecule. This is inconsistent with the experimental data that indicate the majority of 5′-nucleolytic processing events are carried out by the same PolI molecule that has just extended the upstream primer terminus [28].

Thus, our results do not support the model in which PolI performs the two cooperative activities via transition of the flap DNA substrate from the polymerase domain to the 5′-nuclease domain. In other words, the rigid PolI model is an unreasonable one to describe the cooperation of the polymerase and 5′-nuclease domains of PolI so as to leave a nick in the flap DNA substrate.

### Flexible PolI model

In above section, we showed that the rigid PolI model is an unreasonable one to describe the cooperation of PolI's polymerase and 5′-nuclease activities. In this section we examine the feasibility of the flexible PolI model.

#### Model for transition of 5′-nuclease domain from inactive to active modes

It is known that the polymerase domain and the 5′-nuclease domain are connected by a linker of *N* = 16 amino acids [36], [37]. As a reasonable approximation, we consider that the linker behaves like a polymer coil, acting as an “entropic” spring [38]. As will be shown later (see next section), the spring constant of this “entropic” spring is calculated to be 8.56 pN/nm, which is in good agreement with the measured value of about 8.5 pN/nm [39]. Thus, it is considered that the connection between the 5′-nuclease and the polymerase domains is only via the flexible linker and other types of the interaction between the two domains such as electrostatic force, van der Waals force, etc., can be negligible. The “entropic” force of the flexible linker dictates the relative equilibrium positions of the domains as observed in the X-ray determined three-dimensional structure [36]. It must be borne in mind that this is a static “snapshot” but provides us with a starting point, which is shown schematically in Figure 1A. Another hypothesis states that the 5′-nuclease domain has a high affinity for the flap DNA substrate having no single-stranded gap (Figure 1B), as in the rigid PolI model (see above section).

**Figure 4:**
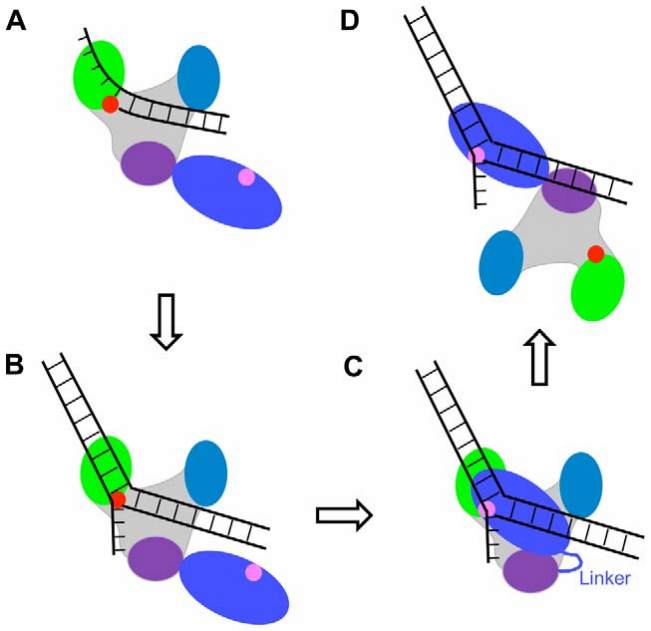
Schematic illustrations of the model for collaboration of the polymerase activity by the polymerase domain and the 5′-nuclease activity by the 5′-nuclease activity (see text for detailed description).

Based on the above hypotheses, the model for coordination between polymerase and 5′-nuclease activities via transition of the 5′-nuclease domain from the inactive to active modes is presented as follows. We begin with the polymerase active site positioned at the replication fork, with the downstream 5′-flap being very far away from the 5′-nuclease domain (Figure 4A). In this case, due to the thermal noise, although the 5′-nuclease domain fluctuates around its equilibrium position, the 5′-nuclease domain cannot reach the 5′-flap. As polymerization proceeds, the 5′-flap becomes closer and closer to the polymerase. Consider now that the polymerase active site is positioned at the fork of the flap (Figure 4B). Via stretching the linker coil and rotating the 5′-nuclease domain, the thermal noise can occasionally usher the active site of the 5′-nuclease domain towards the position adjacent to the polymerase active site. Thus, the 5′-nuclease domain can interact with the flap DNA (Figure 4C). In Figure 4B or 4C, the polymerase has inserted all the complementary nucleotides onto the growing strand that can be incorporated and carried out some strand-displacement synthesis, effectively lifting the 5′ end of the downstream DNA away from its complement. The affinity of the polymerase domain for this structure is not high because it has no free 3′ ssDNA adjacent to the last added nucleotide, i.e., it has synthesized a transient flap. The polymerase domain has lost its grip on the 3′ ssDNA of the substrate [40]–[42] and, now, only the interaction with the dsDNA of the substrate is present. Thus, the polymerase domain now does not show high affinity for the DNA substrate [40]. On the other hand, the 5′-nuclease domain has highest affinity for the flap DNA substrate having no single-stranded gap [28]. Thus, in Figure 4C, the stronger interaction of the 5′-nuclease domain with the DNA substrate expels the polymerase domain detaching from the DNA substrate (Figure 4D), since the 5′-nuclease domain now occupies the same region on the DNA substrate as the polymerase domain did, as seen in Figure 4C. Then, the 5′-flap is cleaved by the 5′-nuclease active site, resulting in a readily ligatable nick with juxtaposed 5′-phospahte and 3-hydroxyl groups.

#### Dynamics for transition of 5′-nuclease domain from inactive to active modes

First, we determine the magnitude of the spring constant resulting from “entropic” spring of the linker coil. The spring constant can be simply calculated by [35]

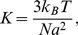
(11)where *a* is the average size of an amino acid. With *k_B_*T = 4.11 pN

nm (*T* = 298 K), *N* = 16 and *a* = 0.3 nm, we obtain from Eq. (11) that *K* = 8.56 pN/nm, which is in good agreement with the experimental value of about 8.5 pN/nm [39]. Thus, throughout our calculations in this section, except in Figure S8 where *K* is varied, we take *K* = 8.56 pN/nm. The good agreement between the calculated and experimental values of *K* implies that the connection between the 5′-nuclease domain and the polymerase domain is only via the flexible linker and other types of the interaction between the two domains such as electrostatic force, van der Waals force, etc., can be negligible.

**Figure 5:**
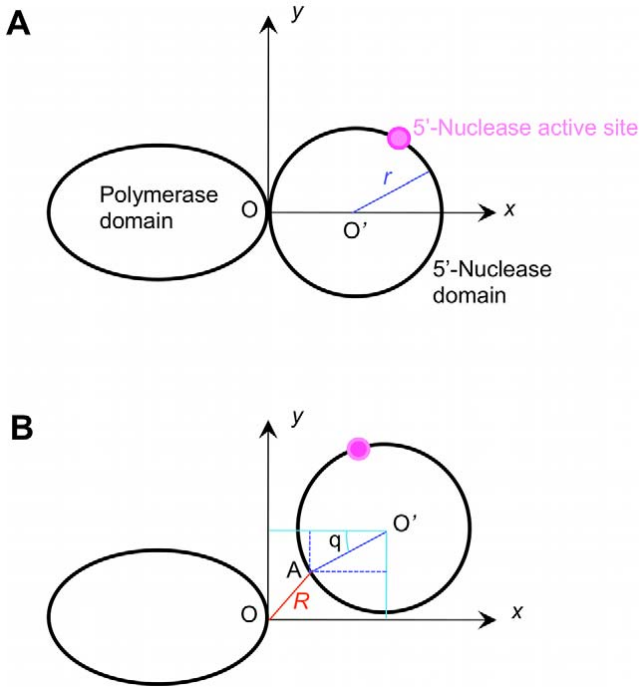
Schematic diagram to illustrate the elastic force of the stretched linker acting on the 5′-nuclease domain as a function of its center-of-mass position (*x*, *y*) and rotation angle *θ*. For approximation, the 5′-nuclease domain is considered as a sphere of radius *r*. O' denotes the center-of-mass position of the 5′-nuclease domain. O denotes the connection point of the linker to the polymerase domain and is taken as the origin of the coordinate O*xy*, while A denotes the connection point of the linker to the 5′-nuclease domain, with |OA|  = *R*. (A) Equilibrium position of the 5′-nuclease domain relative to the polymerase domain. (B) A transient position of the 5′-nuclease domain.

To simplify our analysis of the effect of the elastic force of the stretched linker on the movement and rotation of the 5′-nuclease domain, we approximate the 5′-nuclease domain as a sphere of radius *r* = 3.5 nm (see Figure 5). Here, we only consider the movement of its center-of-mass position in two dimensions (*x*, *y*), where the coordinate O*xy* is shown in Figure 5. For convenience, we take the O*xy* plane here to be the same as that shown in Figure 2 but with different positions of origin O. As we have checked, the inclusion of the movement in *z* direction that is perpendicular to the paper surface in Figure 5 nearly has no effect on our statistical results presented in this section. Thus, the movement of the 5′-nuclease domain relative to the polymerase domain is characterized by its center-of-mass position (*x*, *y*) while the rotation by its rotation angle *θ*. At equilibrium position of the 5′-nuclease domain relative to the polymerase domain, i.e., when the linker is not stretched, the center-of-mass position and rotation angle of the 5′-nuclease domain is written as (*x*, *y*, *θ*)  =  (*r*, 0, 0) (Figure 5A). When the center-of-mass of the 5′-nuclease domain is positioned at (*x*, *y*) and the domain is rotated counterclockwise by an angle of *θ*, the linker is stretched by a length of *R* =  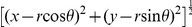 (see Figure 5B). Thus, the elastic force acting on the 5′-nuclease domain along the *x* and *y* directions are 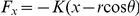 and 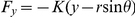, respectively. From the available structure [36], it is noted that, when the 5′-nuclease active site becomes coincident with the polymerase active site, the center-of-mass position and rotation angle of the 5′-nuclease domain can be approximately written as (*x*, *y*, *θ*)  =  (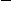 *r*, *d*, *π*), as schematically seen in Figure 4C, where *d* should be in the range of 0<*d*<3 nm. Here, we take *d* as a variable parameter in our calculations. Similar to Eq. (1), the interaction potential of the 5′-nuclease domain with the flap DNA substrate can be written as follows
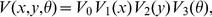
(12)where *V*_0_ is the interaction strength. *V*_1_(*x*), *V*_2_(*y*) and *V*_3_(*θ*) have the following forms
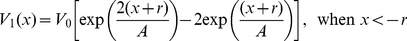
(13)

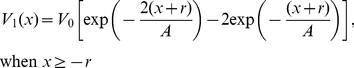
(14)

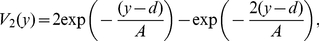
(15)

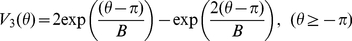
(16)where *rB* = *A* = 0.5 nm.

Thus, the movement and rotation of the 5′-nuclease domain in viscous solution can be described by the following Langevin equations
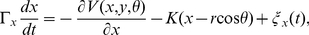
(17)

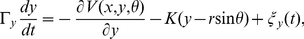
(18)

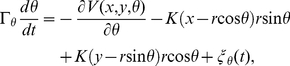
(19)where it is required that 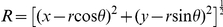 ≤ *Na*  = 4.8 nm; 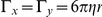  = 6.59
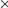
10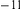 kg

s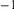 and 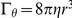  = 1.0766
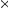
10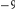 nm^2^ 
kg

s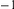; 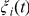 (*i* =  *x*,*y*,*θ*) satisfies 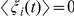, 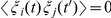 (*i ≠ j*) and 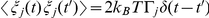. The initial and final conditions for Eqs. (17)–(19) are (*x*, *y*,*θ*)  =  (*r*, 0, 0) and (*x*, *y*,*θ*)  =  (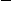 *r*, *d*,*π*), respectively.

**Figure 6:**
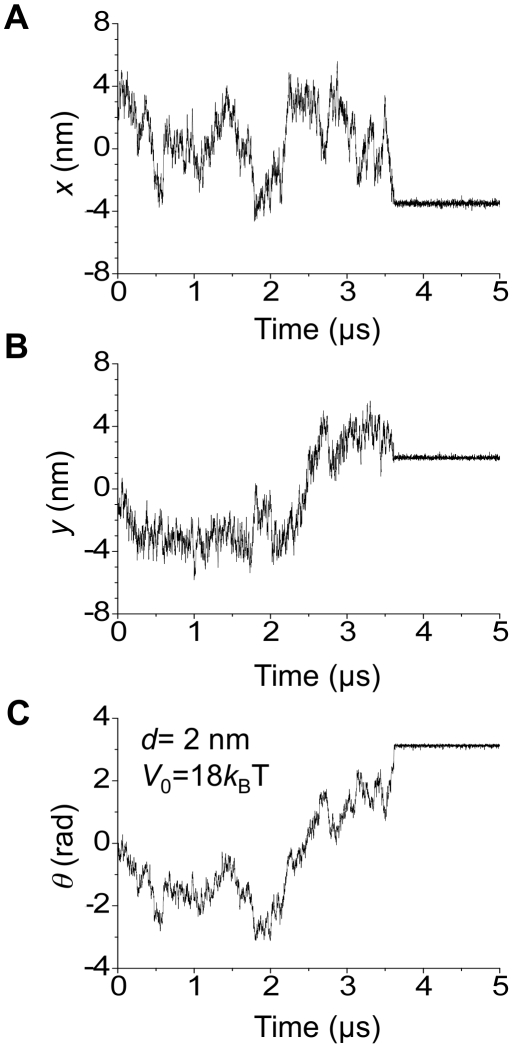
A typical example of the calculated results for the trace of the movement and rotation of the 5′-nuclease domain, with initial position (*x*, *y*,*θ*)  =  (3.5 nm, 0, 0) and final position (*x*, *y*,*θ*)  =  (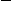
3.5 nm, 2 nm, 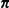).

We solved Eqs. (17)–(19) numerically by using the stochastic Runge-Kutta method, as mentioned above. The calculated results show that the mean time for the 5′-nuclease domain to transit from the inactive to active modes, i.e., the mean time for the 5′-nuclease domain to transit from the initial state (*x*, *y*,*θ*)  =  (*r*, 0, 0) to the final state (*x*, *y*,*θ*)  =  (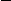 *r*, *d*, *π*) is insensitive to the value of the interaction strength *V*_0_ between the 5′ nuclease domain and the flap DNA substrate (Figure S9). A typical example of results for the trace of the movement and rotation of the 5′-nuclease domain is shown in Figure 6. Two distributions of the transition time are shown in Figure S10. It is seen that the 5′-nuclease domain rapidly transits from the inactive to active modes and the transition time approximately has a single-exponential distribution.

**Figure 7:**
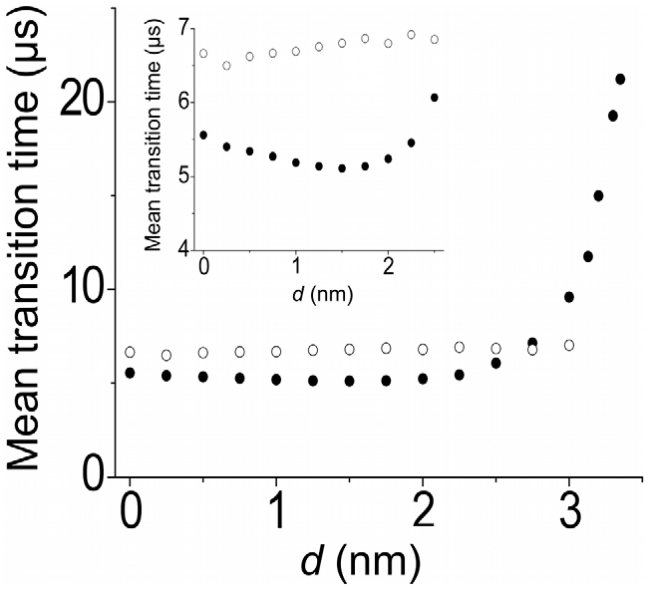
Calculated results of the mean transition time *T_m_* versus *d*. Filled dots represent results with spring constant *K* = 8.56 pN/nm, while unfilled dots represent results with *K* = 0. Inset is the enlargement. *V*_0_ = 18 *k*_B_T.

In Figure 7, we show the calculated results of the mean transition time, *T_m_*, versus *d* (filled dots). It is seen that *T_m_* increases with the increase of *d* when *d*>2.5 nm. Even for the large value of *d*, e.g., *d* = 3 nm, *T_m_* is only about 10 µs, which is a very short time. On the other hand, from Figure 3B it is seen that, when the interaction strength of the polymerase domain with the DNA substrate *U*_0_ is only larger than 13 *k_B_T*, the mean dissociation time of the DNA from the polymerase is *T_d_*>10 µs. In other words, if *U*_0_ is only larger than 13 *k_B_*T, the 5′-nuclease domain has a large probability to bind the flap DNA strongly before its dissociating from the polymerase domain. The affinity of 13 *k_B_*T corresponds to a mean dissociation constant larger than 1 µM, which is a very weak affinity between proteins and DNA. Available experimental data indicated that the polymerase domain binds dsDNA (corresponding to our case as schematically shown in Figure 4B) with a mean dissociation constant of about 100 nM [40], which is equivalent to a binding affinity of about 16 *k_B_*T that is larger than 13 *k_B_*T. In fact, from Figure 3B we see that, at *U*_0_ = 16 *k_B_*T, the mean dissociation time *T_d_*>133 µs that is much larger than *T_m_* = 10 µs. Thus, our results imply that the 5′-nuclease domain has a very large probability to bind the flap DNA strongly before its dissociating from the polymerase domain. In other words, the majority of 5′-nucleolytic processing events are carried out by the same PolI molecule that has just extended the upstream primer terminus, which is consistent with the experimental data [28]. Therefore, we conclude that in PolI the 5′-nuclease domain transits from its equilibrium position to the position near the polymerase active site rather than the flap DNA substrate transits from the polymerase active site to the 5′-nuclease active site. In other words, the flexible PolI model is a reasonable one to describe the collaboration of the polymerase and 5′-nuclease domains of PolI so as to leave a nick in the flap DNA substrate.

Moreover, it is interesting to note from Figure 7 (see the inset) that the minimum value of the mean transition time *T_m_* does not occur at *d* = 0. Rather, *T_m_* decreases slightly with the increase of *d* when *d*≤1.5 nm and the minimum value of *T_m_* occurs at the optimal value of *d*≈1.5 nm. These results, together with the comparison with the results without the elastic force resulting from the “entropic” spring of the linker coil (unfilled dots in Figure 7), imply that the presence of an appropriate elastic force can facilitate the transition of the 5′-nuclease domain to the active mode. This can also be noted from Figure S8, where we show the results of *T_m_* versus the spring constant *K* for different values of *d*. It is seen from Figure S8 that the minimum value of *T_m_* for *d* = 2 nm occurs at *K* = 8.56 pN/nm, while *T_m_* around *K* = 8.56 pN/nm for *d* = 1.5 nm and *d* = 2.5 nm have very small values that are near the minimum values. Taken together, it is expected from these results that, in order for the 5′-nuclease domain to have the most efficient transition from the inactive to active modes, *d* is about 1.5∼2 nm.

**Figure 8:**
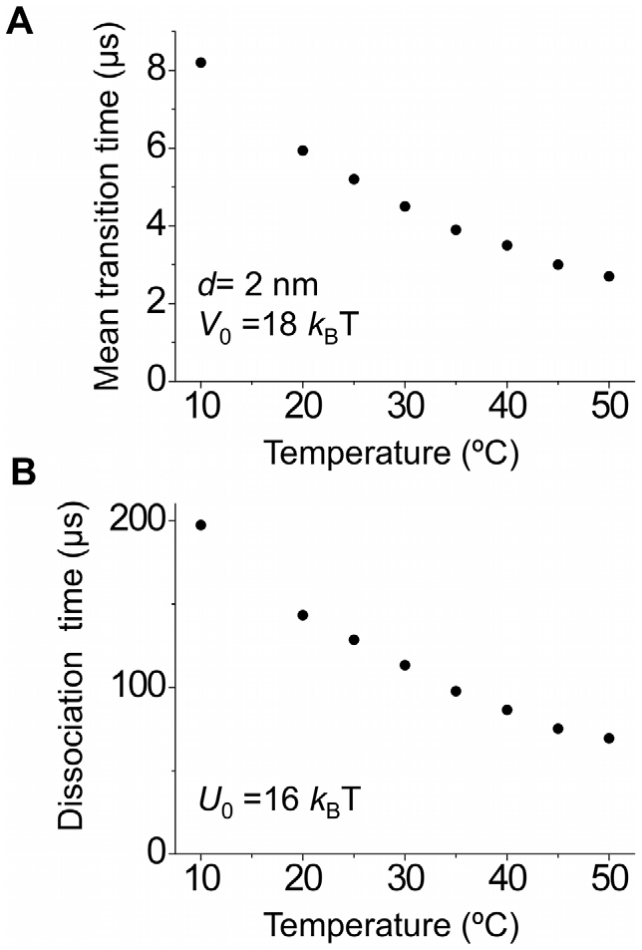
Effect of temperature. (A) Calculated results of the mean transition time *T_m_* of the 5′-nuclease domain to the active mode as a function of the temperature. (B) Calculated results of the mean dissociation time *T_d_* of the flap DNA substrate from the polymerase domain as a function of the temperature.

In the above, we have fixed temperature T = 298 K (25°C). To see the effect of the variation of temperature on the results, we change the temperature in our calculations. The value of viscosity *η* as a function of the temperature is taken from the experimental data (see Table S1 and [43]). The calculated results of the mean time *T_m_* for the 5′-nuclease domain to transit to the active mode as a function of the temperature for *d* = 2 nm are shown in Figure 8A. It is seen that, as the temperature increases, the mean transition time *T_m_* decreases, which results from both the decrease of the viscosity *η* and the increase of the noise strength. On the other hand, the statistical results of the mean time *T_d_* for the flap DNA with a nick to dissociate from the polymerase domain as a function of the temperature are shown in Figure 8b, where we take *U*_0_ = 16 *k_B_*T that is consistent with the available experimental data [40] (see above). As expected, the mean dissociation time *T_d_* decreases as the temperature increases. By comparing Figure 8A with Figure 8B, it is seen that, at a given temperature, *T_d_* is much larger than *T_m_*, implying that, at any temperature in the range of 10–50°C, the 5′-nucleolytic processing event is most probably carried out by the same PolI molecule that has just extended the upstream primer terminus.

**Figure 9:**
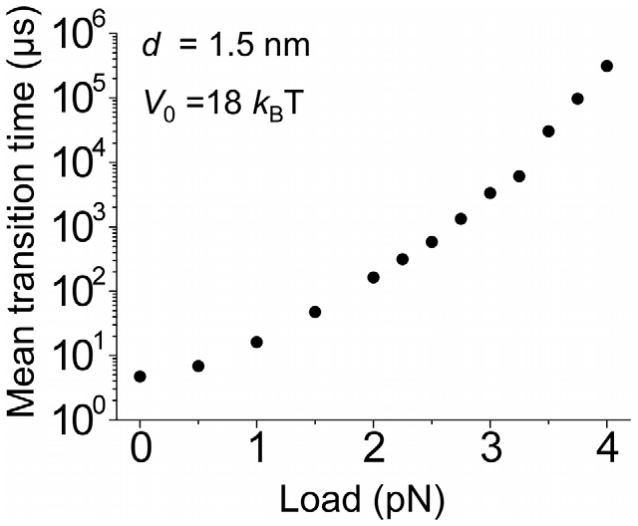
Predicted results of the mean transition time *T_m_* versus the external load *F_load_* acting on the 5′-nuclease domain.

Next, we present our predicted results for the effect of the external force on the transition of the 5′-nuclease domain from the inactive to active modes. Consider a load, *F_load_*, acting on the residues indicated by the blue dot (Figure S11) along the *x* direction. Then, the right-hand sides of Eqs. (17), (18) and (19) should be added by terms 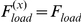, 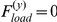, 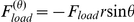, respectively. The calculated results of the mean transition time *T_m_* versus *F_load_* at T = 298 K (25°C) are shown in Figure 9. It is seen that *T_m_* increases significantly with the increase of the external load. It is interesting to note that, only at *F_load_*>2.5 pN, *T_m_*>600 µs that is much larger than *T_d_* = 133 µs at *U*_0_ = 16 *k_B_*T (see above). This implies that the flap DNA substrate becomes dissociated from the polymerase before the 5′-nuclease domain transits to the active mode. Thus, the polymerase molecule that has just extended the upstream primer terminus cannot perform the activity of cleaving the downstream 5′-flap, i.e., the polymerase cannot simultaneously perform the two activities.

## Discussion

In this work, our theoretical results indicate that, to perform the 5′-nuclease activity, it is reasonable that the 5′-nuclease domain transits from its equilibrium position to the position near the polymerase active site (the flexible PolI model) rather than the flap DNA substrate transits from the polymerase active site to the 5′-nuclease active site (the rigid PolI model). By contrast, during the proofreading process, it is reasonable that the DNA transits between the polymerase active site and the 3′ – 5′ exonuclease active site. In detail, in order to cleave the mismatched base, the 3′ – 5′ single-stranded DNA is argued to transit from the polymerase active site to the exonuclease active site by unwinding some base pairs [44].

With the flexible PolI model, the difference in the position of the 5′-nuclease domain relative to the polymerase domain in the crystal structure of *Taq* polymerase bound to the inhibitory Fab observed by Urs *et al*. [45] from that without the inhibitory Fab observed by Kim *et al*. [36] can be easily explained as follows. Consider that the inhibitory Fab has a high binding affinity for the polymerase domain, with the interacting surface located near the contacting region with the 5′-nuclease domain when it is in the equilibrium position relative to the polymerase domain. As our results showed, the thermal noise can easily drive the 5′-nuclease domain away from its equilibrium position. Thus, it is expected that, once the 5′-nuclease domain deviates away from its equilibrium position, the inhibitory Fab can binds to the polymerase domain. The occupancy of the inhibitory Fab prevents the 5′-nuclease domain from returning to its equilibrium position. This is consistent with the observed crystal structure of *Taq* polymerase bound to the inhibitory Fab [44].

The balanced action of DNA PolI's 5′ nuclease and polymerase activities is not only important *in vivo*. It is also of particular importance in the widely used Taqman, or fluorogenic 5′ nuclease assay [46]. These assays are used in a plethora of diagnostic and research activities [47], [48]. In the 5′ nuclease assay, reporter and quencher dyes are placed at either end of an oligonucleotide designed to hybridize to a specific target DNA sequence to be detected. This produces a fluorogenic probe. Once hybridized to the target, this probe can be degraded by the 5′ nuclease activity of *Taq* polymerase and is detected by an increase in fluorescence that occurs on separation of the reporter and quencher dyes. This leads to loss of Forster resonance energy transfer (FRET) between the donor and quencher dyes and an increase in signal at the emission wavelength of the fluorophore. When a fragment containing this target DNA is subjected to PCR amplification using Taq polymerase with flanking primers, the target probe is cleaved by the 5′ nuclease activity provided the probe is fully annealed to its target. If a mismatch is present within the otherwise complementary sequence, the polymerase activity simply displaces the probe rather than hydrolyzing it. Thus, understanding the mechanisms of 5′ nuclease/polymerase coordination may be useful in designing more efficient 5′ nuclease assays.

Finally, it is mentioned that, to test the flexible PolI model, it is hoped to experimentally determine the prediction that, when an external force *F_load_*>2.5 pN acts on the 5′-nuclease domain, the polymerase molecule that has just extended the upstream primer terminus cannot perform the activity of cleaving the downstream 5′-flap (see Figure 9).

## Supporting Information

Figure S1 Experimentally observed x-ray structure of *Taq* polymerase based on 1TAQ.pdb. Thumb (light blue), palm (grey), fingers (green), proofreading domain (purple), and 5′ -nuclease domain (dark blue) are shown as a backbone cartoon rendered using Pymol (DeLano Scientific). An oversized grey sphere marks the active site of the 5′-nuclease domain, while red spheres provide reference to the position occupied by dCTP in the active site of the polymerase (modeled from 5KTQ.pdb). The distance between these two features is indicated in Å.
(TIF)

Figure S2 Forms of interaction potential of the polymerase domain and the 5′-nuclease domain with the flap DNA substrate, *U* (*x*, 0, 0), *U* (0, *y*, 0) and *U* (0, 0, *z*), with *A* = 0.5 nm and *U*_0_ = *k*_B_T.
(TIF)

Figure S3 A typical result for the trace of DNA relative to the DNA polymerase. *U*_0_ = 16 *k*_B_T.
(TIF)

Figure S4 A typical result for the trace of DNA relative to the DNA polymerase. *U*_0_ = 16 *k*_B_T.
(TIF)

Figure S5 A typical result for the trace of DNA relative to the DNA polymerase. *U*_0_ = 16 *k*_B_T.
(TIF)

Figure S6 A typical result for the trace of DNA relative to the DNA polymerase. *U*_0_ = 16 *k*_B_T.
(TIF)

Figure S7 Another form of the interaction potential of the polymerase domain and the 5′-nuclease domain with the flap DNA substrate, with *U* (*x*, 0, 0), *U* (0, *y*, 0) and *U* (0, 0, *z*) being shown in (A), (B) and (C), respectively. *U*_0_ = *k*_B_T.
(TIF)

Figure S8 Calculated results of the mean transition time *T_m_* versus the spring constant *K* for different values of *d*. Dotted line corresponds to *K* = 8.56 pN/nm. *V*_0_ = 18 *k*_B_T
(TIF)

Figure S9 Calculated results of the mean time *T_m_* for the 5′-nuclease domain to transit from the inactive to active modes as a function of the interaction strength *V*_0_ between the 5′-nuclease domain and the flap DNA substrate, with *d* = 2 nm.
(TIF)

Figure S10 Time distributions of the 5′-nuclease domain transiting from the inactive to active modes. (A) *d* = 2 nm. (B) *d* = 2.5 nm.
(TIF)

Figure S11 Schematic diagram to illustrate the external load *F_load_* acting on the residues (blue dots) of the 5′-nuclease domain. (A) Equilibrium position of the 5′-nuclease domain relative to the polymerase domain. (B) A transient position of the 5′-nuclease domain.
(TIF)

Table S1 Temperature dependence of viscosity.
(DOC)
