# Supplementary figures and images for: A Model for Transition of 5′-Nuclease Domain of DNA Polymerase I from Inert to Active Modes
Source: PLoS One. 2011 Jan 14;6(1):e16213. doi: 10.1371/journal.pone.0016213 (PMC3021548; doi:10.1371/journal.pone.0016213)

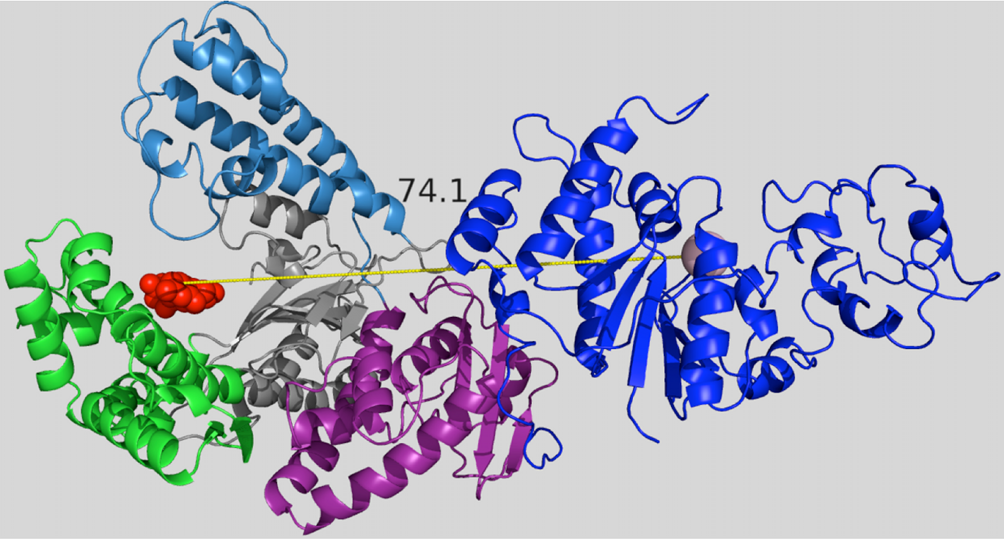

Supplement: Figure S1 — Experimentally observed x-ray structure of Taq polymerase based on 1TAQ.pdb. Thumb (light blue), palm (grey), fingers (green), proofreading domain (purple), and 5′ -nuclease domain (dark blue) are shown as a backbone cartoon rendered using Pymol (DeLano Scientific). An oversized grey sphere marks the active site of the 5′-nuclease domain, while red spheres provide reference to the position occupied by dCTP in the active site of the polymerase (modeled from 5KTQ.pdb). The distance between these two features is indicated in Å. (TIF) [file pone.0016213.s001.tif]

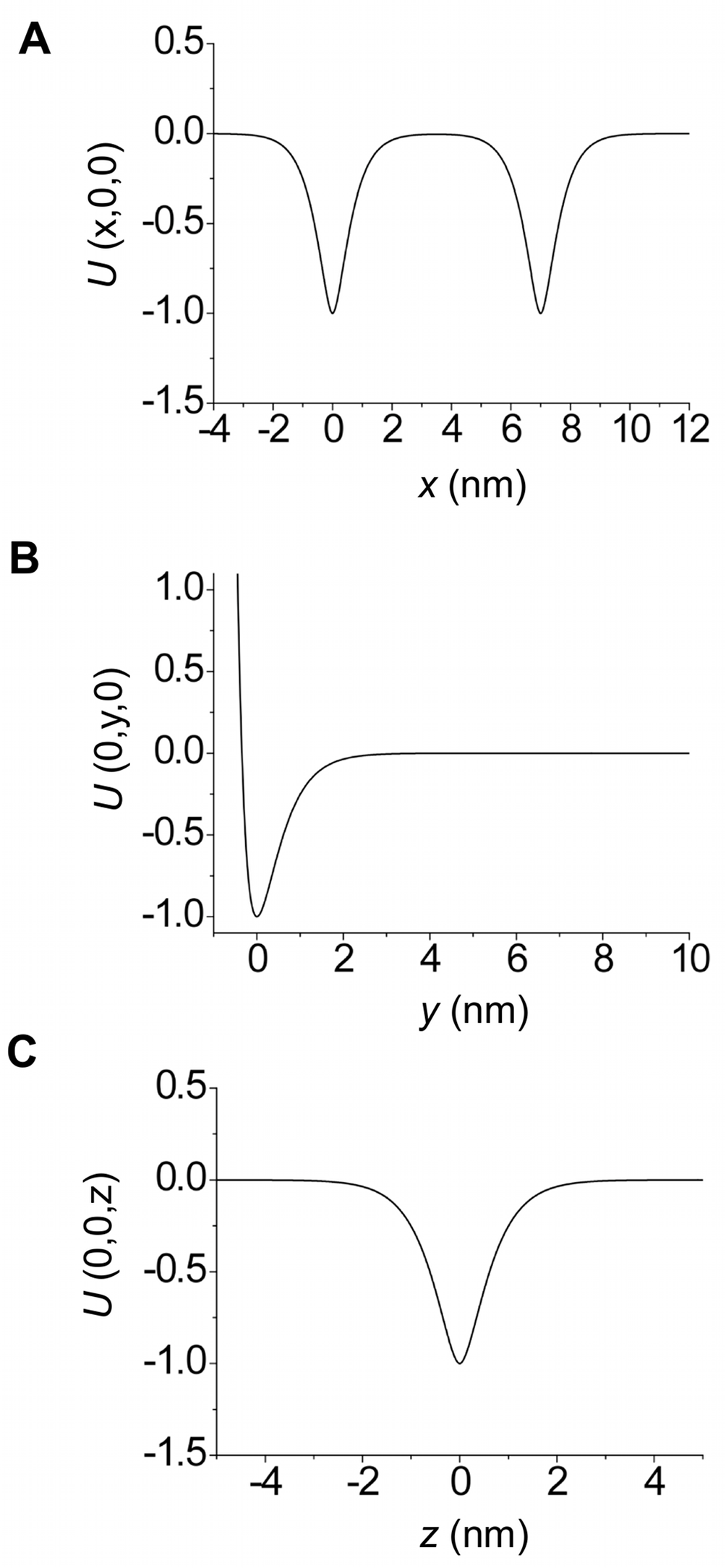

Supplement: Figure S2 — Forms of interaction potential of the polymerase domain and the 5′-nuclease domain with the flap DNA substrate, U (x, 0, 0), U (0, y, 0) and U (0, 0, z), with A = 0.5 nm and U 0 = k BT. (TIF) [file pone.0016213.s002.tif]

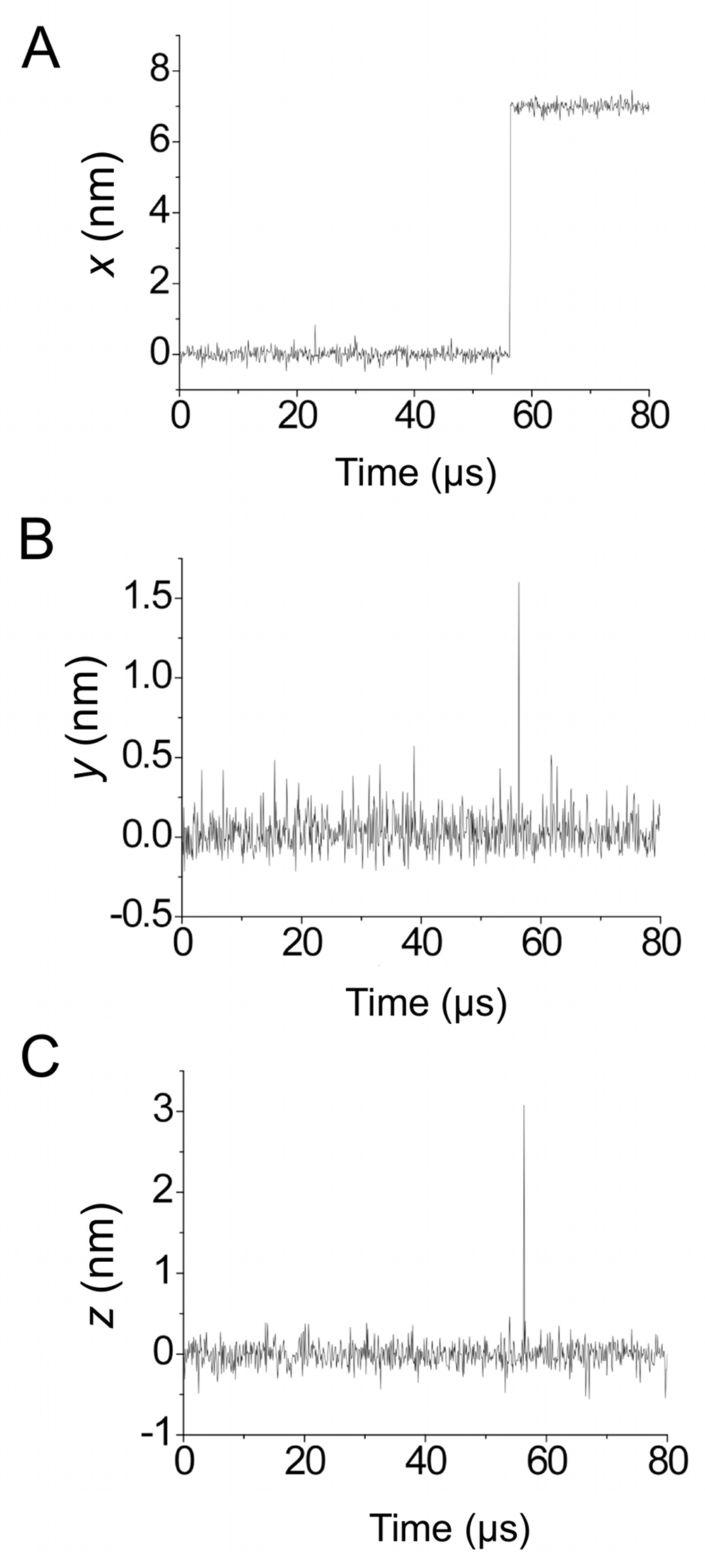

Supplement: Figure S3 — A typical result for the trace of DNA relative to the DNA polymerase. U 0 = 16 k BT. (TIF) [file pone.0016213.s003.tif]

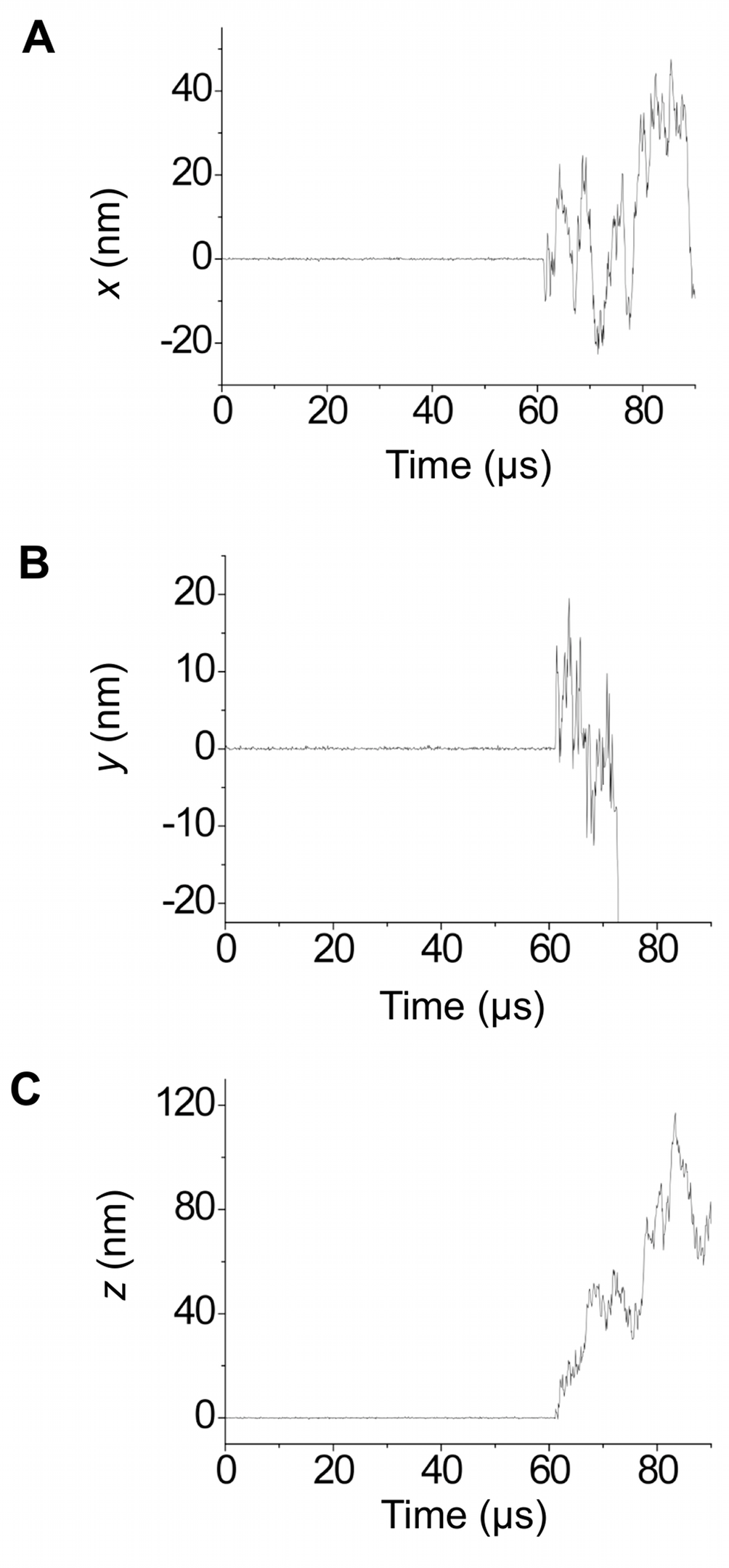

Supplement: Figure S4 — A typical result for the trace of DNA relative to the DNA polymerase. U 0 = 16 k BT. (TIF) [file pone.0016213.s004.tif]

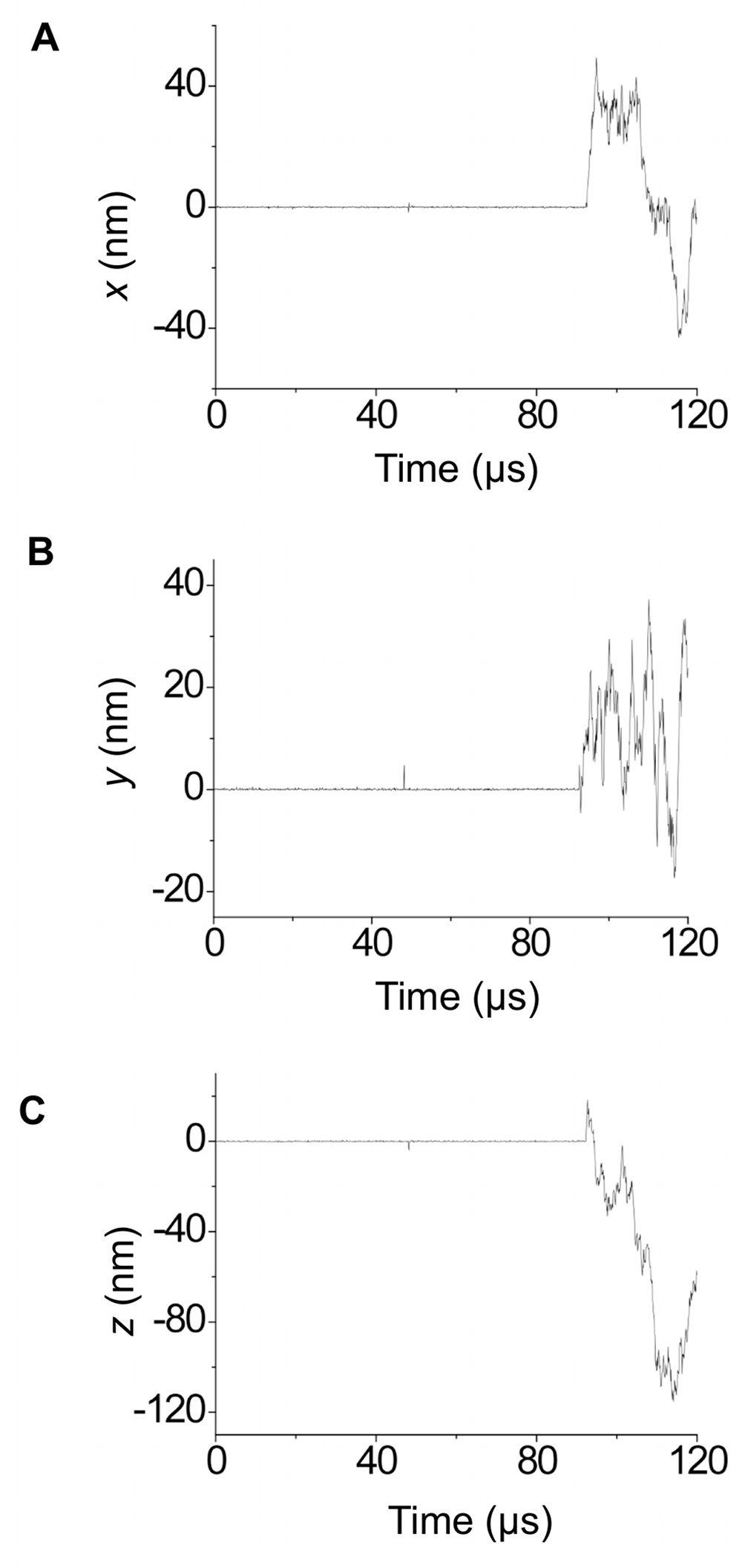

Supplement: Figure S5 — A typical result for the trace of DNA relative to the DNA polymerase. U 0 = 16 k BT. (TIF) [file pone.0016213.s005.tif]

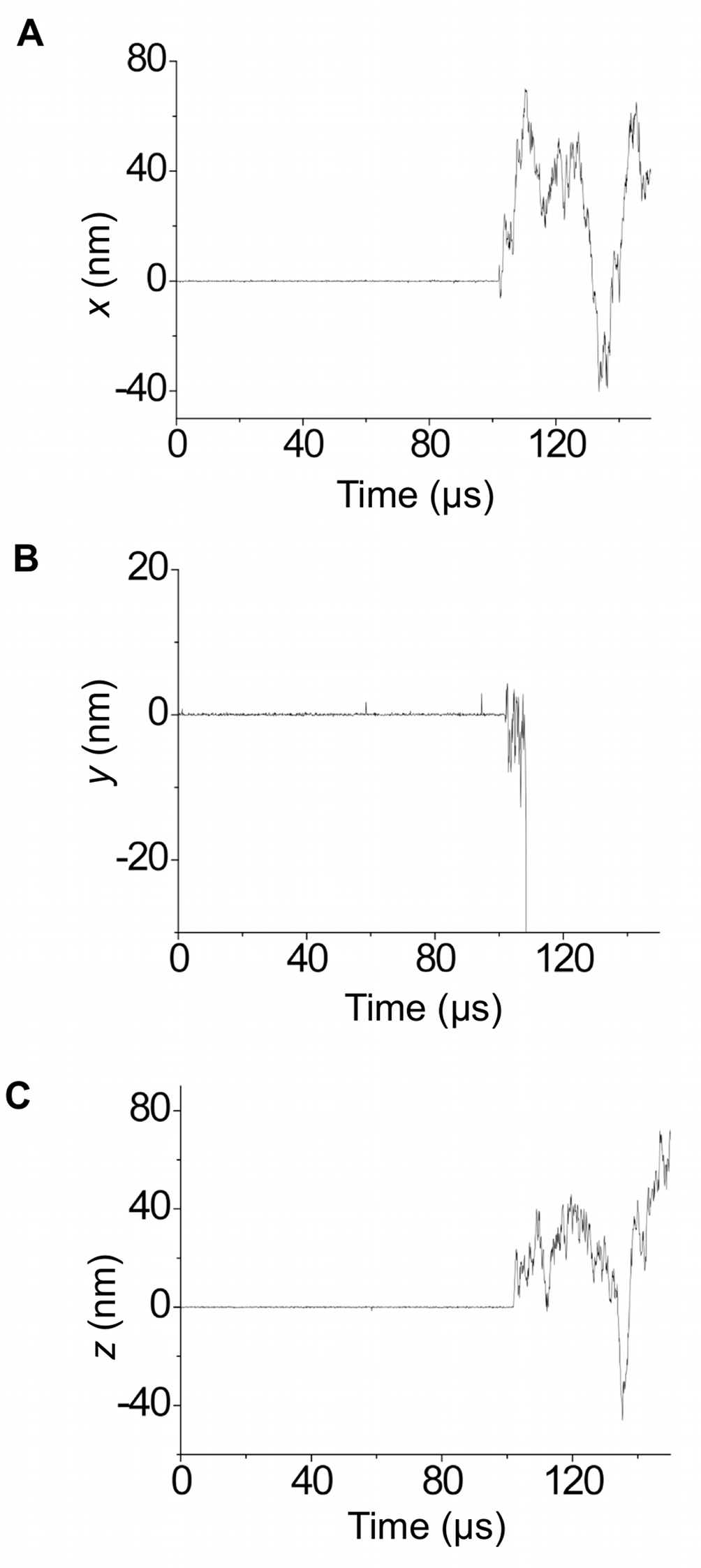

Supplement: Figure S6 — A typical result for the trace of DNA relative to the DNA polymerase. U 0 = 16 k BT. (TIF) [file pone.0016213.s006.tif]

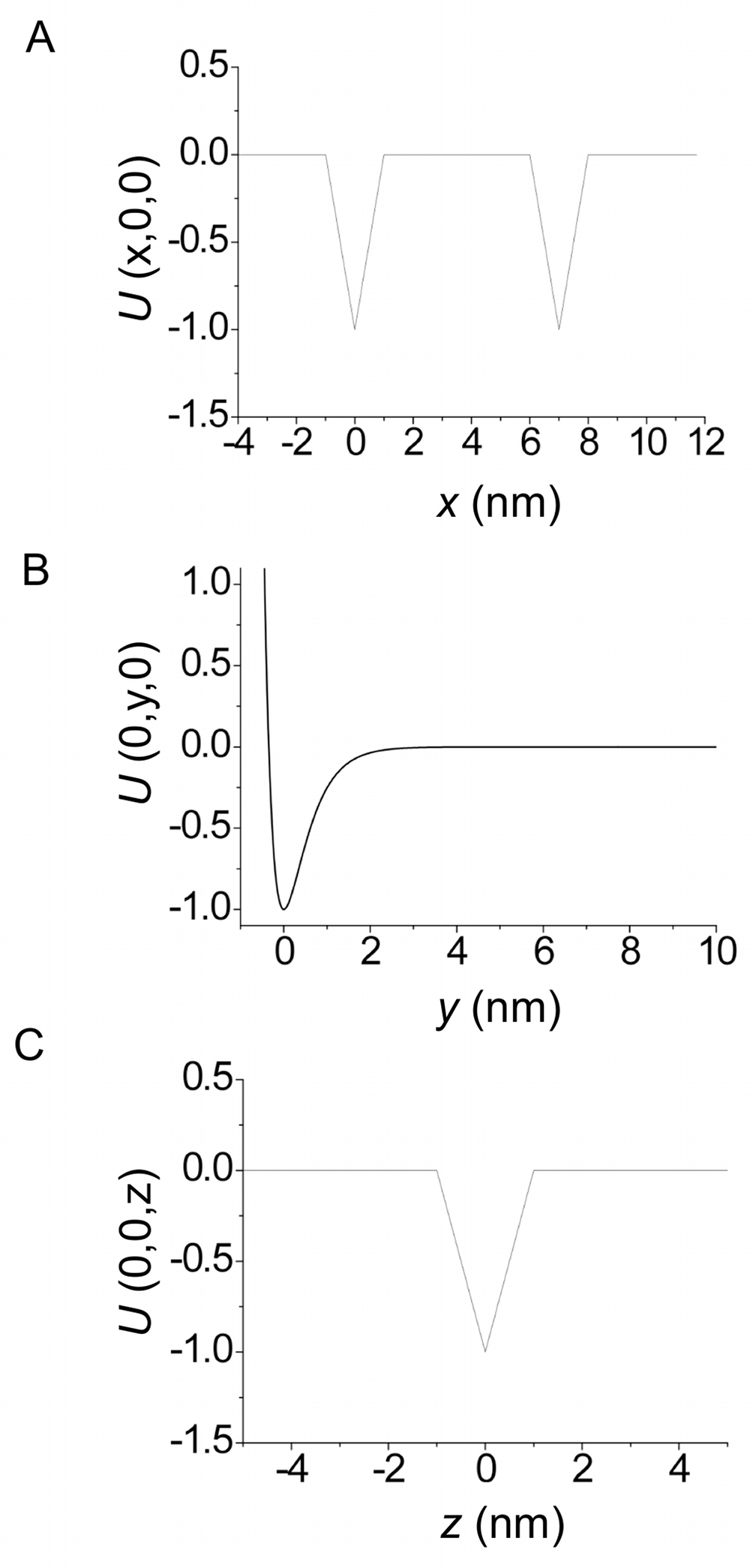

Supplement: Figure S7 — Another form of the interaction potential of the polymerase domain and the 5′-nuclease domain with the flap DNA substrate, with U (x, 0, 0), U (0, y, 0) and U (0, 0, z) being shown in (A), (B) and (C), respectively. U 0 = k BT. (TIF) [file pone.0016213.s007.tif]

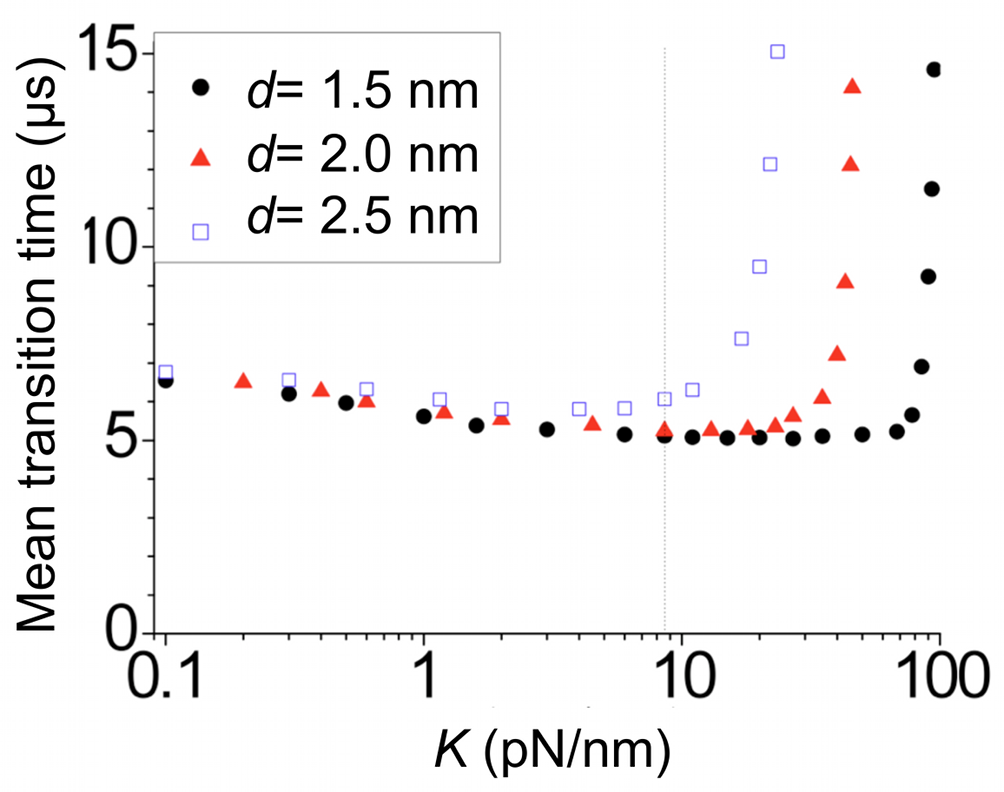

Supplement: Figure S8 — Calculated results of the mean transition time Tm versus the spring constant K for different values of d. Dotted line corresponds to K = 8.56 pN/nm. V 0 = 18 k BT (TIF) [file pone.0016213.s008.tif]

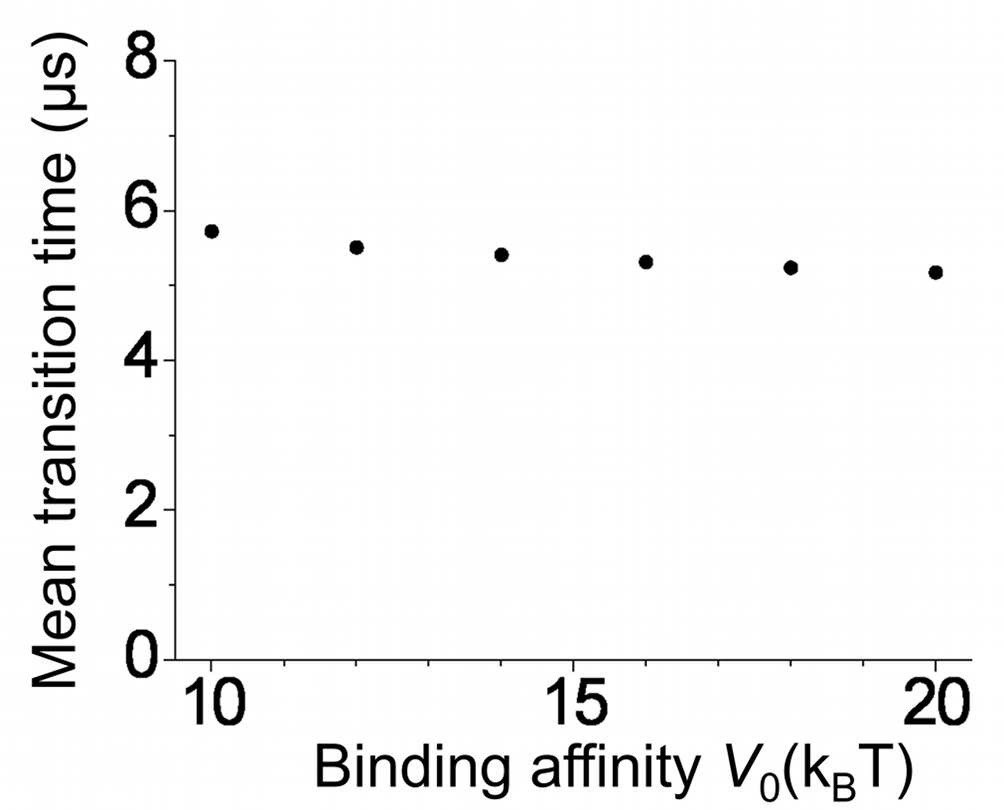

Supplement: Figure S9 — Calculated results of the mean time Tm for the 5′-nuclease domain to transit from the inactive to active modes as a function of the interaction strength V 0 between the 5′-nuclease domain and the flap DNA substrate, with d = 2 nm. (TIF) [file pone.0016213.s009.tif]

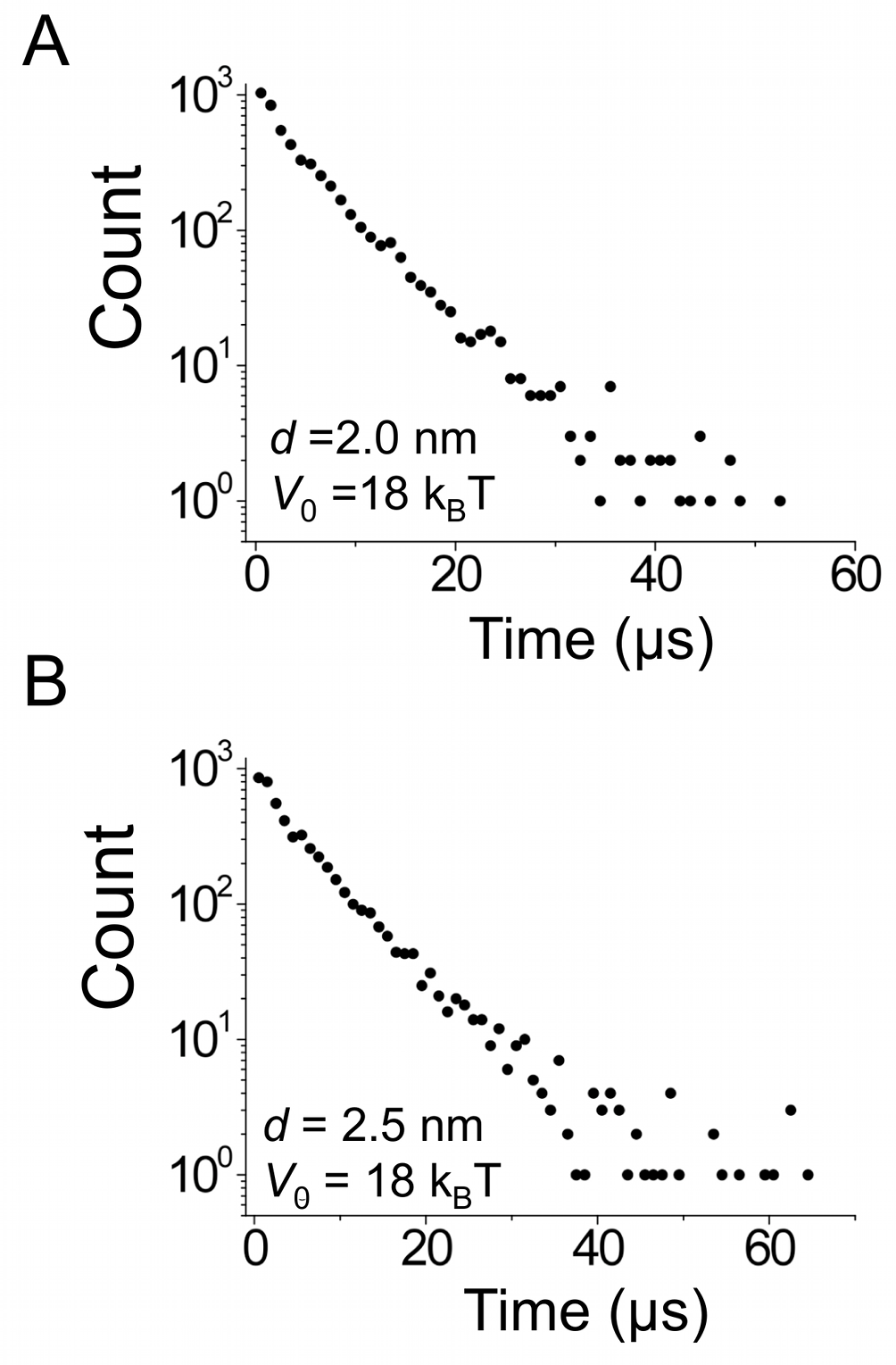

Supplement: Figure S10 — Time distributions of the 5′-nuclease domain transiting from the inactive to active modes. (A) d = 2 nm. (B) d = 2.5 nm. (TIF) [file pone.0016213.s010.tif]

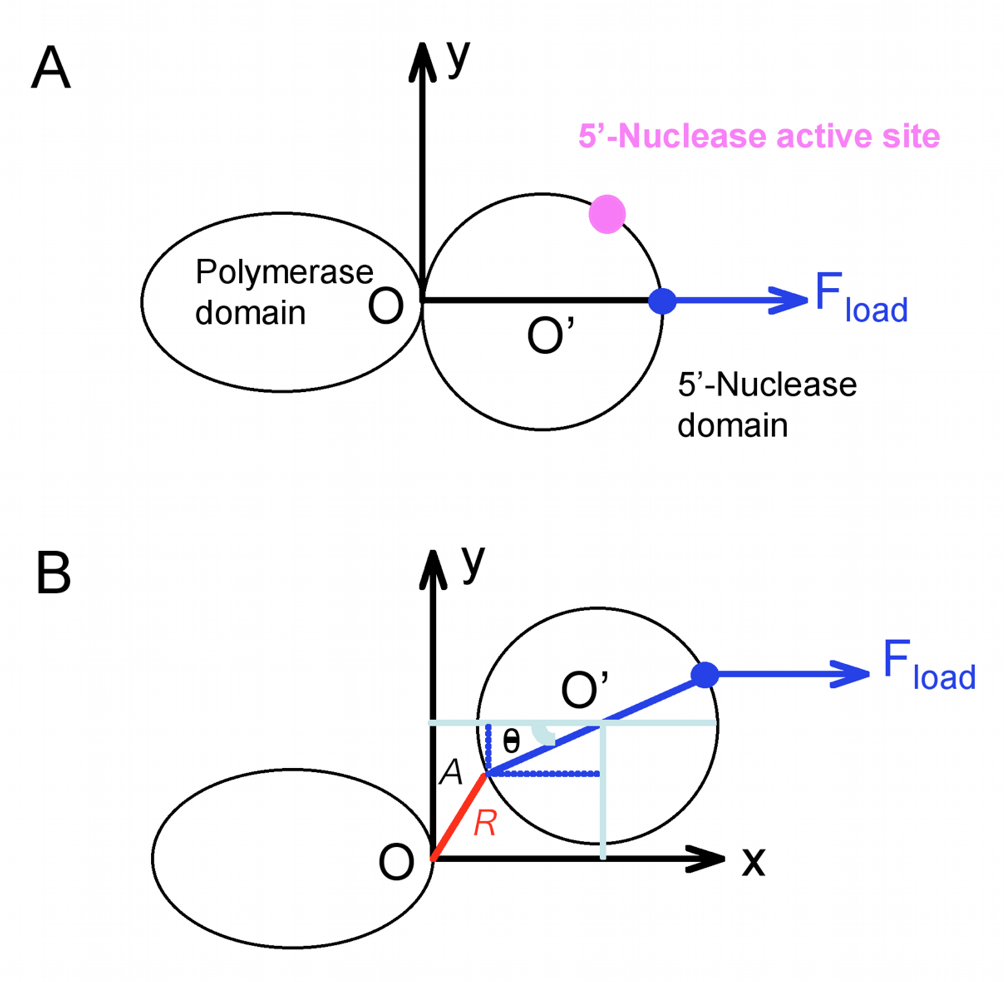

Supplement: Figure S11 — Schematic diagram to illustrate the external load Fload acting on the residues (blue dots) of the 5′-nuclease domain. (A) Equilibrium position of the 5′-nuclease domain relative to the polymerase domain. (B) A transient position of the 5′-nuclease domain. (TIF) [file pone.0016213.s011.tif]
