# Supplementary material for: A Model for Transition of 5′-Nuclease Domain of DNA Polymerase I from Inert to Active Modes
Source: PLoS One. 2011 Jan 14;6(1):e16213. doi: 10.1371/journal.pone.0016213 (PMC3021548; doi:10.1371/journal.pone.0016213)
Supplement: Table S1 — Temperature dependence of viscosity. (DOC) [file pone.0016213.s012.doc]

Temperature () 10 20 25 30 35 40 45 50

_____________________________________________________________________Viscosity (gms) 1.3 1 0.892 0.799 0.722 0.656 0.598 0.549

_____________________________________________________________________
